# Outcomes of COVID-19 and Influenza in Cerebral Palsy Patients Hospitalized in the United States: Comparative Study of a Nationwide Database

**DOI:** 10.3390/v16081284

**Published:** 2024-08-12

**Authors:** Mohammed A. Quazi, Muhammad Hassan Shakir, Zohaa Faiz, Ibrahim Quraishi, Adeel Nasrullah, Hafiz Abdullah Ikram, Amir H Sohail, Sulaiman Sultan, Abu Baker Sheikh

**Affiliations:** 1 Department of Mathematics and Statistics, University of New Mexico, Albuquerque, NM 87106, USA; maquazi@salud.unm.edu; 2 Department of Internal Medicine, The Wright Center for Graduate Medical Education, Scranton, PA 18505, USA; shakirm@thewrightcenter.org; 3 Department of Medicine, School of Medicine, Aga Khan University, Karachi 74000, Pakistan; zohaa.faiz22@gmail.com; 4 Department of Internal Medicine, University of New Mexico, Albuquerque, NM 87106, USAhaikram@unm.edu (H.A.I.); ssultan@salud.unm.edu (S.S.); 5 Division of Pulmonology and Critical Care, Allegheny Health Network, Pittsburg, PA 15212, USA; adeel.nasrullah@ahn.org; 6 Division of Surgical Oncology, University of New Mexico, Albuquerque, NM 87106, USA; asohail@salud.unm.edu

**Keywords:** cerebral palsy, COVID-19, influenza, mortality, in-hospital outcomes

## Abstract

Patients with cerebral palsy (CP) are particularly vulnerable to respiratory infections, yet comparative outcomes between COVID-19 and influenza in this population remain underexplored. Using the National Inpatient Sample from 2020–2021, we performed a retrospective analysis of hospital data for adults with CP diagnosed with either COVID-19 or influenza. The study aimed to compare the outcomes of these infections to provide insights into their impact on this vulnerable population. We assessed in-hospital mortality, complications, length of stay (LOS), hospitalization costs, and discharge dispositions. Multivariable logistic regression and propensity score matching were used to adjust for confounders, enhancing the analytical rigor of our study. The study cohort comprised 12,025 patients—10,560 with COVID-19 and 1465 with influenza. COVID-19 patients with CP had a higher in-hospital mortality rate (10.8% vs. 3.1%, *p* = 0.001), with an adjusted odds ratio of 3.2 (95% CI: 1.6–6.4). They also experienced an extended LOS by an average of 2.7 days. COVID-19 substantially increases the health burden for hospitalized CP patients compared to influenza, as evidenced by higher mortality rates, longer hospital stays, and increased costs. These findings highlight the urgent need for tailored strategies to effectively manage and reduce the impact of COVID-19 on this high-risk group.

## 1. Introduction

In the context of the ongoing COVID-19 pandemic, assessing its impact on individuals with pre-existing neurological disorders, particularly cerebral palsy (CP), is critical. CP, the most prevalent motor disability in childhood, increases susceptibility to respiratory pathogens, a concern magnified by the emergence of SARS-CoV-2 and influenza [1]. Advancements in medical care have significantly improved survival for individuals with CP, with life expectancy for those with mild cerebral palsy now mirroring the general population up to the age of 58 [2,3]. This evolution extends the scope of CP into adulthood, challenging the historical view of it as predominantly a pediatric condition [4].

CP individuals have a unique clinical profile that influences their response to respiratory infections, necessitating a specialized approach to their care during pandemics [3,4,5]. Barriers to accessing appropriate healthcare arising from systemic, clinician-related, and personal challenges accentuate the urgency of our analysis [4,5]. This study examines the outcomes of COVID-19 in CP patients compared to those hospitalized with influenza, focusing on clinical outcomes, healthcare resource utilization, and mortality. We aim to illuminate the differential impact of COVID-19 and influenza on this vulnerable cohort, providing indispensable insights to guide the development of tailored healthcare interventions for CP patients during current and future respiratory pandemics.

## 2. Materials and Methods

A retrospective analysis was conducted using data from the 2020–2021 National Inpatient Sample (NIS), including 10,560 COVID-19 patients diagnosed with cerebral palsy. Additionally, 1465 CP patients diagnosed with influenza were identified for comparison. Our study utilized multivariate regression analysis and propensity score matching to evaluate in-hospital outcomes such as mortality rates, comorbidity profiles, healthcare expenditures, duration of hospital stays, and discharge disposition, comparing those with CP and COVID-19 to CP patients with influenza.

The NIS database, a comprehensive repository of anonymized billing and diagnostic information from participating hospitals across the United States, was selected for its extensive coverage of hospitalizations, offering a robust dataset for this comparative study [6]. Given the database’s reliance on de-identified data, our research was exempt from institutional review board oversight in compliance with federal regulations [7]. Inclusion criteria were limited to individuals with CP aged 18 and above hospitalized with COVID-19 or influenza, identified through specific ICD-10 CM and procedure codes, excluding cases with co-infections or incomplete data. All included variables were identified using ICD codes, which are provided in Supplementary Table S1.

The NIS dataset provided comprehensive insights into in-hospital outcomes, procedures, and discharge data. This study organized the variables into three categories: patient level, hospital level, and illness severity. At the patient level, variables included age, race, gender, comorbidities, insurance status, income based on zip code, and discharge outcomes. Hospital-level variables comprised the facility’s location, teaching status, bed size, and geographical region. Variables pertaining to illness severity included length of hospital stay (LOS), mortality rate, hospitalization costs, the Elixhauser comorbidity index, and specific in-hospital complications such as mechanical ventilation, use of vasopressors, tracheostomy, cardiac arrest, cardiac dysrhythmias, acute venous thromboembolism (VTE), acute kidney injury (AKI), AKI requiring hemodialysis, and seizures.

Python programming language was employed for data analysis and statistical modeling, while data curation was conducted using SAS. The chi-square test for independence was employed to assess the relationships between categorical variables in the two cohorts, as presented in Table 1. Small *p*-values indicate a non-independence between the variables. For instance, a significant relationship exists between gender and cohort variables (*p*-value < 0.001). Simple linear regression was utilized to determine independent variables (*p* ≤ 0.2) for continuous outcomes, such as LOS and total charges, which were then used to construct a multivariate regression model. Similarly, logistic regression was applied to identify independent variables (*p* ≤ 0.2) for binary outcomes like invasive mechanical ventilation and AKI. Additionally, a secondary analysis on a propensity-matched sample (PSM) was conducted to validate the initial statistical model findings, addressing the imbalance in sample sizes between the COVID-19 and CP group and the influenza and CP control group.

## 3. Results

Table 1 presents a comparative analysis of patients diagnosed with COVID-19 and those with Influenza. The cohort includes 12,025 patients, with a larger proportion being COVID-19 positive (10,560 cases, 87.8%) as opposed to Influenza positive (1465 cases, 12.2%).

In terms of gender, the data does not show a significant difference between COVID-19 and Influenza patients (males: 58.6% vs. 60.8%, *p* = 0.111). The mean age for females with COVID-19 is marginally higher than that for Influenza (51.6 years vs. 46.8 years), and a similar trend is observed among males (51.2 years vs. 42.9 years). The difference in age distribution across groups is statistically significant, particularly in the 18–29 age category (14.3% for COVID-19 vs. 27.0% for Influenza, *p* < 0.001). The racial distribution reveals significant discrepancies, with Black and Hispanic individuals more frequently affected by COVID-19 than Influenza (Black: 17.2% vs. 14.7%, Hispanic: 9.7% vs. 14.0%, *p* < 0.001). Median household income does not differ significantly between the two diseases (lowest bracket: 30.7% for COVID-19 vs. 29.7% for Influenza, *p* = 0.494). Insurance status shows a notable difference; a greater fraction of Influenza patients rely on Medicaid in comparison to those with COVID-19 (33.4% vs. 24.6%, *p* < 0.001). Hospital characteristics such as division and bed size also show significant differences (for large bed size hospitals: 45.6% for COVID-19 vs. 47.1% for Influenza, *p* = 0.007). The prevalence of certain comorbidities significantly differs, with a higher occurrence in COVID-19 patients compared to those with Influenza (e.g., hypertension: 42.0% vs. 31.4%, *p* = 0.046; obesity: 15.6% vs. 8.2%, *p* < 0.001; chronic pulmonary disease: 18.3% vs. 25.9%, *p* < 0.001; depression: 12.8% vs. 6.8%, *p* < 0.001; dementia: 4.8% vs. 1.0%, *p*< 0.001).

In patients with CP and COVID-19, the odds of in-hospital mortality were significantly higher compared to those with CP and influenza, as determined through multivariable logistic regression (10.8% vs. 3.1%, *p* = 0.001), with an adjusted odds ratio (aOR) of 3.2 (95% CI: 1.6–6.4), as detailed in Table 2. This finding was substantiated by a PSM analysis, which indicated a similarly increased mortality risk in the COVID-19 cohort, with an aOR of 3.1 (95% CI: 1.6–6.3, *p* < 0.007), presented in Table 3.

Table 2 compares the in-hospital outcomes between the CP and COVID-19 group and the CP and influenza group. The analysis revealed no statistically significant differences in the risk of acute kidney injury (18.9% vs. 13%; aOR: 1.3, 95% CI: 0.9–1.9, *p* = 0.14), use of vasopressors (2.9% vs. 2.4%; aOR: 1.2, 95% CI: 0.5–2.6, *p* = 0.71), invasive mechanical ventilation (13.4% vs. 11.6%; aOR: 1.25, 95% CI: 0.8–1.8, *p* = 0.27), seizures (43.8% vs. 55.6%; aOR: 0.8, 95% CI: 0.6–1.04, *p* = 0.1), tracheostomy (1% vs. 2%; aOR: 0.6, 95% CI: 0.2–1.5, *p* = 0.28), and venous thromboembolism (VTE) (4% vs. 2.4%; aOR: 1.7, 95% CI: 0.8–3.7, *p* = 0.18). These findings persisted, as reflected in the post-PSM analysis in Table 3. However, the propensity-matched analysis highlighted lower risks in certain complications for the CP and COVID-19 patients: non-invasive mechanical ventilation (3.8% vs. 7.1%; aOR: 0.5, 95% CI: 0.2–0.99, *p* = 0.048) and cardiac dysrhythmias (2.7% vs. 6.5%; aOR: 0.4, 95% CI: 0.2–0.8, *p* = 0.02), as shown in Table 3.

CP patients with COVID-19 exhibited a longer average length of stay (LOS), at 9.5 days, compared to those with influenza, who averaged 7.2 days. This difference persisted after PSM, with COVID-19 patients having adjusted higher length of stay of 2.7 days (*p* < 0.004) compared to influenza patients. Furthermore, the inflation-adjusted average hospitalization cost for COVID-19 patients was USD 24,478, exceeding that of the influenza group, which was USD 19,338; this results in a marked cost differential of USD 4960 post-PSM (*p* < 0.04). The increased length of stay (LOS) for COVID-19 patients with cerebral palsy, averaging 2.7 days longer than influenza patients, indicates a higher burden on healthcare resources and potentially greater patient morbidity. Additionally, the higher hospitalization costs for COVID-19 patients reflect the increased intensity of care required, which may include more frequent interventions, extended monitoring, and higher utilization of critical care services. These findings underscore the need for targeted healthcare strategies to manage and allocate resources effectively for this vulnerable population during pandemics. Regarding discharge disposition, patients with COVID-19 were more likely to be transferred to short-term acute care facilities rather than being routinely discharged, compared to their influenza counterparts Table 2 and Table 3.

## 4. Discussion

In this retrospective observational study, we present the first comprehensive comparison of outcomes between COVID-19 and influenza infections in adult patients with cerebral palsy. To our knowledge, this investigation encompasses the largest cohort of hospitalized CP patients evaluated for disparities in outcomes related to these infections. Previous studies have evaluated respiratory infections in CP patients; however, there is a substantial gap in our understanding of COVID-19’s impact [8]. Our analysis indicates that individuals with cerebral palsy experience higher inpatient mortality from COVID-19 compared to influenza. The demographics of our study population predominantly consist of males, with over 65% of participants in both groups being White. Notably, conditions such as hypertension, diabetes mellitus, obesity, depression, and dementia were more prevalent among the COVID-19 patients, while chronic pulmonary disease was more common in the influenza group. Moreover, the influenza group demonstrated higher rates of non-invasive ventilation and experienced cardiac dysrhythmias more frequently compared to their COVID-19 counterparts.

Age and gender differences in our study align with previously reported trends in the general population, where a higher proportion of both COVID-19 and influenza groups consisted of males [9]. Studies involving the general population have consistently indicated not only poorer outcomes from COVID-19 in men but also a higher susceptibility to contracting the virus [10,11]. These differences may be attributed to several factors: higher rates of symptomatic disease and subsequent detection in men, higher smoking rates among men, and gender-based genetic differences in immune response to pathogens [12,13,14]. Previously published data suggested that hospitalized patients with COVID-19 are generally younger than those with influenza, although this difference was not statistically significant in our study [9,15]. However, the literature indicates that patients with cerebral palsy face a greater risk of hospitalization from COVID-19 compared to those without CP, with the effect being more pronounced among males [16]. Moreover, we observed Blacks and Hispanics more frequently affected by COVID-19. Pertaining research indicates that these communities face disproportionate rates of infection and severe outcomes due to socioeconomic disparities, occupational exposure, limited healthcare access, and higher prevalence of comorbidities (Price-Haywood et al., 2020; Rubin-Miller et al., 2020; Tai et al., 2021). These factors collectively contribute to the observed disparities in COVID-19 impact [17,18,19].

Although no prior studies have directly compared the outcomes of COVID-19 and influenza in patients with cerebral palsy and other disabilities, our results are consistent with general population trends [20,21]. Our cerebral palsy cohort shows a COVID-19 case fatality rate of 11%, compared to a pooled 15% in the general hospitalized population [22], and notably higher than general population mortality [16]. The CDC reports that influenza case fatality rates varied widely from 4% to 40% during the 2019–2020 season, with a decrease likely due to enhanced public health measures and increased influenza vaccination during the early COVID-19 pandemic [23,24]. Moreover, COVID-19 patients face higher risks of needing mechanical ventilation and ICU care, although this study found no statistical difference [25]. Test positivity and hospitalization rates for influenza declined significantly, with comprehensive data on case fatality rates still limited [26]. Increased influenza vaccination, associated with lower mortality and less severe disease, likely improved outcomes [27,28]. Patients with cerebral palsy are at greater risk due to factors like aspiration risk, recurrent infections, and compromised pulmonary function. Many reside in communal living facilities, increasing exposure and transmission risks [29]. The Advisory Committee on Immunization Practices emphasizes vaccinating individuals with disabilities against influenza due to worse outcomes [29]. Despite similar ARDS incidence in both groups, ARDS presents more severe COVID-19 cases [30,31]. Non-US studies also show higher ICU mortality rates for COVID-19 [32]. This analysis highlights the significant impact of respiratory viruses on vulnerable populations and the need for specialized public health strategies. Our study highlights distinct comorbidity profiles between COVID-19 and influenza patients. For COVID-19, obesity, diabetes, and hypertension are major mortality risk factors, while heart failure, renal failure, chronic respiratory failure, and malignancy predominate in influenza-related deaths [33]. Despite adjusting for these conditions, COVID-19 mortality rates were significantly higher, suggesting that factors beyond comorbidities, such as the virus’s virulence and lack of an early specific vaccine, contribute to its severity. This emphasizes the need for tailored clinical strategies that consider each patient’s unique comorbidity profile and the interactions between their health conditions and the infection. Furthermore, cerebral palsy patients with COVID-19 experienced longer hospital stays, higher healthcare costs, and increased transfers to acute care, indicating more severe clinical impacts and resource demands compared to those with influenza.

Our research advances our understanding of how respiratory infections affect hospitalized cerebral palsy patients, demonstrating greater morbidity and healthcare use than seen in the general population. Yet, it also uncovers unanswered questions vital for future research, such as how cerebral palsy intensifies the severity of these infections. Future studies should examine the influence of individual comorbidities on treatment outcomes and the efficacy of specific treatments for this population. The effects of COVID-19 variants and the long-term consequences of infections on recovery and quality of life also require thorough investigation. Addressing these issues will fill critical knowledge gaps and improve clinical guidelines and patient care protocols.

Our study, utilizing the NIS and based on ICD-10 coding, faces limitations typical of retrospective analyses from administrative databases. Specifically, the potential for errors in coding practices can introduce inaccuracies in identifying diagnoses, thereby affecting the reliability of our data. Additionally, the lack of detailed clinical data, such as vaccination status, laboratory results, imaging, treatment used, and longitudinal follow-up, restricts our ability to assess patient trajectories and outcomes comprehensively. Furthermore, our findings are confined to inpatient experiences and do not capture outpatient trends or long-term effects, which may differ significantly. It is also important to note that this analysis predates the widespread availability of COVID-19 vaccines, potentially limiting the applicability of our conclusions to current hospitalization and treatment trends.

## 5. Conclusions

This study offers the first detailed comparison of in-hospital outcomes for cerebral palsy patients affected by COVID-19 versus influenza, revealing that COVID-19 leads to significantly higher mortality, longer hospital stays, and greater healthcare costs. These findings highlight the harsh impact of COVID-19 on this vulnerable group and underscore the need for specialized healthcare strategies to manage and reduce risks. Despite controlling for various factors, COVID-19′s inherent severity results in increased resource use and poorer outcomes, emphasizing the importance of continued research into effective treatments and preventive measures, such as vaccination. Future research should include longitudinal data and explore the effects of COVID-19 vaccinations to enhance clinical practices and care quality for these patients.

## Figures and Tables

**Table 1:** Patient-level characteristics for COVID-19-positive patients with cerebral palsy and Influenza-positive patients with cerebral palsy.

Characteristics	CP and COVID-19+		CP and Influenza+		*p*-Value
	**N**	**%**	**N**	**%**	
N = 12,025	10,560	87.82	1465	12.18	
**Gender (%)**	**N**	**%**	**N**	**%**	0.111
Female	4375	41.43	575	39.25	
Male	6185	58.57	890	60.75	
**Mean Age Years (SD)**	**Mean**	**SD**	**Mean**	**SD**	
Female	51.61	17.62	46.77	18.75	
Male	51.16	16.75	42.88	16.97	
**AGE Groups (%)**	**N**	**%**	N	%	**<0.001**
18–29	1505	14.25	395	26.96	
30–49	2985	28.27	455	31.06	
50–69	4495	42.57	470	32.08	
≥70	1575	14.91	145	9.9	
**RACE (%)**	**N**	**%**	**N**	**%**	**<0.001**
Asian or Pacific Islander	160	1.52	30	2.05	
Black	1815	17.19	215	14.68	
Hispanic	1025	9.71	205	13.99	
Native American	85	0.8	*	*	
Other	350	3.31	55	3.75	
White	7125	67.47	960	65.53	
**MEDIAN HOUSEHOLD INCOME (%)**	**N**	**%**	**N**	**%**	0.494
≤51,999	3245	30.73	435	29.69	
52,000–65,999	2890	27.37	420	28.67	
66,000–87,999	2495	23.63	330	22.53	
≥88,000	1930	18.28	280	19.11	
**INSURANCE STATUS (%)**	**N**	**%**	**N**	**%**	**<0.001**
Medicaid	2600	24.62	490	33.45	
Medicare	6920	65.53	830	56.66	
Other	135	1.28	*	*	
Private Insurance	825	7.81	125	8.53	
Self-pay	80	0.76	*	*	
**HOSPITAL DIVISION (%)**	**N**	**%**	**N**	**%**	**<0.001**
East North Central	2030	19.22	265	18.09	
East South Central	590	5.59	35	2.39	
Middle Atlantic	1880	17.8	235	16.04	
Mountain	520	4.92	115	7.85	
New England	550	5.21	95	6.48	
Pacific	1185	11.22	195	13.31	
South Atlantic	1990	18.84	215	14.68	
West North Central	775	7.34	135	9.22	
West South Central	1040	9.85	175	11.95	
**HOSPITAL BED SIZE (%)**	**N**	**%**	**N**	**%**	**0.007**
Large	4810	45.55	690	47.1	
Medium	3125	29.59	465	31.74	
Small	2625	24.86	310	21.16	
**HOSPITAL TEACHING STATUS (%)**	**N**	**%**	**N**	**%**	0.306
Rural	1110	10.51	145	9.9	
Urban nonteaching	1785	16.9	270	18.43	
**COMORBIDITIES (%)**	**N**	**%**	**N**	**%**	***p*-value**
HTN	4430	41.95	460	31.4	0.046
Diabetes	2050	19.41	160	10.92	<0.001
Cancer	185	1.75	30	2.05	0.423
Obesity	1645	15.58	120	8.19	<0.001
Drug Misuse	80	0.76	*	*	0.755
Smoking	800	7.58	100	6.83	0.306
Alcohol	70	0.66	N/A	N/A	0.93
Chronic Pulmonary Disease	1930	18.28	380	25.94	<0.001
Hypothyroidism	1675	15.86	245	16.72	0.398
Autoimmune	160	1.52	*	*	0.141
Depression	1355	12.83	100	6.83	<0.001
AIDS	30	0.28	*	*	0.703
Dementia	505	4.78	*	*	<0.001

AIDS: acquired immunodeficiency syndrome, HTN: hypertension, *: data suppressed.

**Table 2:** In-hospital outcomes for CP with COVID-19-positive and CP with Influenza-positive cohorts.

Complications	COVID-19 N (%)	Influenza N (%)	Odds Ratio for COVID-19+	95% CI Lower Limit	95% CI Upper Limit	*p*-Value
Sudden Cardiac Arrest	215 (2.04)	**	2.00	0.61	6.57	0.256
Vasopressor Use	310 (2.94)	35 (2.39)	1.16	0.52	2.59	0.712
Acute Kidney Injury (AKI)	1995 (18.89)	190 (12.97)	1.32	0.91	1.91	0.139
Invasive Mechanical Ventilation	1410 (13.35)	170 (11.6)	1.25	0.84	1.85	0.267
Non-Invasive Mechanical Ventilation	610 (5.78)	105 (7.17)	0.78	0.48	1.28	0.322
Hemodialysis	145 (1.37)	20 (1.37)	0.66	0.22	1.98	0.455
Venous Thromboembolism (VTE)	425 (4.02)	35 (2.39)	1.71	0.78	3.74	0.179
Tracheostomy	110 (1.04)	30 (2.05)	0.60	0.24	1.51	0.276
Seizures	4620 (43.75)	815 (55.63)	0.80	0.62	1.04	0.095
Cardiac Dysrhythmia	850 (8.05)	95 (6.48)	0.92	0.55	1.54	0.749
Acute Respiratory Distress Syndrome (ARDS)	535 (5.07)	35 (2.39)	1.96	0.89	4.29	0.094
Mean Inflation-Adjusted Cost ($) *	$23,447.62 *	$19,337.74 *	-	-	-	0.004
Mean Length of Stay (Days) ^	9.46 ^	7.19 ^	-	-	-	<0.001
In Hospital Mortality (N = 1185)	1140 (10.8)	45 (3.07)	3.21	1.62	6.36	0.001
**Disposition**						<0.001
Against Medical Advice	45 (0.43)	**	-	-	-	
Home Health Care	1580 (14.96)	240 (16.38)	-	-	-	
Routine	4185 (39.63)	830 (56.66)	-	-	-	
Transfer Other	3395 (32.15)	310 (21.16)	-	-	-	-
Transfer to a Short-Term Hospital	210 (1.99)	30 (2.05)	-	-	-	-

* Inflation-adjusted cost = $5459.60 higher for COVID-19+; ^ the adjusted length of stay = 2.13 days longer for COVID-19+; ** data suppressed. The adjusted for age, hospital bed size, race, gender, hospital location, hospital teaching status, hospital region, median household income, expected primary payer (insurance status), and Elixhauser comorbidities.

**Table 3:** In-hospital outcomes for CP with COVID-19 positive and CP with Influenza positive cohorts after Propensity score matching.

Complications	COVID-19 N (%)	Influenza N (%)	Odds Ratio for COVID-19+	95% CI Lower Limit	95% CI Upper Limit	*p*-Value
Sudden Cardiac Arrest	20 (1.37)	**	1.23	0.26	5.82	0.798
Vasopressor use	35 (2.39)	35 (2.39)	1.01	0.35	2.94	0.985
Acute Kidney Injury (AKI)	190 (12.97)	190 (12.97)	1.01	0.62	1.65	0.968
Invasive Mechanical Ventilation	225 (15.36)	170 (11.6)	1.36	0.84	2.22	0.210
Non-Invasive Mechanical Ventilation	55 (3.75)	105 (7.17)	0.46	0.21	0.99	0.048
Hemodialysis	**	20 (1.37)	0.57	0.10	3.15	0.517
Venous Thromboembolism (VTE)	45 (3.07)	35 (2.39)	1.46	0.52	4.14	0.471
Tracheostomy	**	30 (2.05)	0.54	0.13	2.20	0.389
Seizures	730 (49.8)	815 (55.63)	0.71	0.50	1.01	0.057
Cardiac Dysrhythmia	40 (2.73)	95 (6.48)	0.35	0.15	0.85	0.020
Acute Respiratory Distress Syndrome (ARDS)	70 (4.78)	35 (2.39)	2.05	0.81	5.18	0.130
Mean Inflation-Adjusted Cost ($) *	$24,058.25 *	$19,337.74 *	-	-	-	0.004
Mean Length of Stay (Days) *	9.95 *	7.19 *	-	-	-	<0.001
In Hospital Mortality (N = 1185)	125 (8.5)	45 (3.07)	2.97	1.35	6.56	0.007
**Disposition**						<0.001
Against Medical Advice	**	**	-	-	-	
Home Health Care	230 (15.7)	240 (16.38)	-	-	-	
Routine	700 (47.78)	830 (56.66)	-	-	-	
Transfer Other	355 (24.23)	310 (21.16)	-	-	-	-
Transfer to a Short-Term Hospital	50 (3.41)	30 (2.05)	-	-	-	-

* Inflation-adjusted cost = $4959.52 higher for COVID-19+; * The adjusted length of stay = 2.7 days longer for COVID-19+; ** data suppressed. The 1:1 matching was adjusted for age, hospital bed size, race, gender, hospital location, hospital teaching status, hospital region, median household income, expected primary payer (insurance status), and Elixhauser comorbidities.

## Data Availability

The data used in this study are from the National (Nationwide) Inpatient Sample for 2020 and 2021, obtained from the Healthcare Cost and Utilization Project. The NIS is a publicly available database, and researchers interested in accessing the data can obtain it directly from the HCUP website (https://www.hcup-us.ahrq.gov/), accessed on 7 March 2024.

## Supplementary Materials

The following supporting information can be downloaded at https://www.mdpi.com/article/10.3390/v16081284/s1, Table S1: ICD10 Clinical Modification Codes; Table S2: Propensity Matched: Baseline Patients Characteristics 1:1 Propensity matched variables: Age, sex, race, income status, region, bed size, teaching status, elihauser comorbidities, and insurance status.
