# Supplementary material for: Outcomes of COVID-19 and Influenza in Cerebral Palsy Patients Hospitalized in the United States: Comparative Study of a Nationwide Database
Source: Viruses. 2024 Aug 12;16(8):1284. doi: 10.3390/v16081284 (PMC11359358; doi:10.3390/v16081284)
Supplement: Supplementary file 1 [file viruses-16-01284-s001.zip › viruses-3137468-Table S1.pdf]

Disease/Procedure	ICD-10 Codes
Cerebral palsy	G800,G801,G802,G803,G804,G808,G809
Mechanical ventilation invasive	0BH17EZ,0BH18EZ,5A1935Z,5A0945Z,5A0955Z,5A1945Z,5A1955Z
Smoking	F17,F172,F1720,F17200,F17201,F17203,F17208,F17209,F1721,F17210,F17211,F17213,F17218,F17219,F1722,F17220,F17221,F17223,F17228,F17229,F1729,F17290,F17291,F17293,F17298,F17299,Z87891
Vasopressor use	3E030XZ,3E033XZ,3E040XZ,3E043XZ,3E050XZ,3E053XZ,3E060XZ,3E063XZ
Non-invasive mechanical ventilation	5A09457,5A09458,5A09358,5A09557,5A09558,5A09357,5A0935Z,5A0945Z,5A0955Z
Sudden cardiac arrest	I462,I468,I469
Hemodialysis	5A1D70Z,5A1D90Z,5A1D80Z,5A1D00Z,5A1D60Z
Tracheostomy	0B110F4,0B110Z4,0B113F4,0B113Z4,0B114F4,0B114Z4
VTE	I82210,I82220,I82290,I82401,I82402,I82403,I82409,I82411,I82412,I82413,I82419,I82421,I82422,I82423,I82429,I82431,I82432,I82433,I82439,I82441,I82442,I82443,I82449,I82451,I82452,I82453,I82459,I82461,I82462,I82463,I82469,I82491,I82492,I82493,I82499,I824Y1,I824Y2,I824Y3,I824Y9,I824Z1,I824Z2,I824Z3,I824Z9,I82601,I82602,I82603,I82609,I82611,I82612,I82613,I82619,I82621,I82622,I82623,I82629,I82890,I8290,I82A11,I82A12,I82A13,I82A19,I82B11,I82B12,I82B13,I82B19,I82C11,I82C12,I82C13,I82C19,I1260,I2601,I2602,I2690,I2692,I2693,I2694,I2699,I2609
Chronic pulmonary disease	Elixhauser comorbidities index
Diabetes (2 types)	Elixhauser comorbidities index
AIDS	Elixhauser comorbidities index
Drug abuse	Elixhauser comorbidities index
Cancer (5 types)	Elixhauser comorbidities index
Dementia	Elixhauser comorbidities index
Autoimmune	Elixhauser comorbidities index
Depression	Elixhauser comorbidities index
Hypothyroidism	Elixhauser comorbidities index
Obesity	Elixhauser comorbidities index
HTN	Elixhauser comorbidities index
Alcohol	Elixhauser comorbidities index
AKI	N170,N171,N172,N178,N179,N990
Seizures	G40001,G40009,G40011,G40019,G40101,G40109,G40111,G40119,G40201,G40209,G40211,G40219,G40301,G40309,G40311,G40319,G40401,G40409,G40411,G40419,G40501,G40509,G40801,G40802,G40803,G40804,G40811,G40812,G40813,G40814,G40821,G40822,

	G40823,G40824,G40833,G40834,G4089,G40901,G40909,G40911, G40919,G40A01,G40A09,G40A11,G40A19,G40B01,G40B09,G40B11, G40B19
Cardiac dysrhythmia	I470,I471,I472,I479,I480,I481,I4811,I4819, I482,I4820,I4821,I483,I484,I4891,I4892,I491, I492,I493,I4940,I4949,I495,I498,I499
G-tube placement	0D16074,0D160J4,0D160K4,0D163J4,0D16474,0D164J4,0D164K4, 0D164Z4,0D160Z4,0D16874,0D168J4,0D168K4,0D168Z4,0D9600Z,0D960ZZ, 0D9640Z,0D964ZZ,0DC60ZZ,0DC63ZZ,0DC64ZZ,0DH603Z
ARDS	J810,J80
Ventilator associated pneumonia	J95851
COVID-19	U071
Influenza A	J09X1,J09X2,J09X3,J09X9
Influenza other	J1000,J101,J1008,J101,J102,J1081, J1082,J1083,J1089,J1100,J1108,J111, J112,J1181,J1182,J1183,J1189

Supplemental Table S1: ICD 10 codes
