# Supplementary material for: Outcomes of COVID-19 and Influenza in Cerebral Palsy Patients Hospitalized in the United States: Comparative Study of a Nationwide Database
Source: Viruses. 2024 Aug 12;16(8):1284. doi: 10.3390/v16081284 (PMC11359358; doi:10.3390/v16081284)
Supplement: Supplementary file 1 [file viruses-16-01284-s001.zip › viruses-3137468-Table S2.pdf]

Characteristics	Covid-19+		Influenza+		P-value
	N	%	N	%	
N = 2,930	1465	50.00	1465	50.00	
Gender (%)	N	%	N	%	0.038
Female	630	43	575	39.25	
Male	835	57	890	60.75	
Mean Age Years (SD)	Mean	SD	Mean	SD	
Female	44.09	18.41	46.77	18.75	
Male	43.66	16.99	42.88	16.97	
AGE Groups (%)	N	%	N	%	0.222
18-29	395	26.96	395	26.96	
30-49	500	34.13	455	31.06	
50-69	425	29.01	470	32.08	
>=70	145	9.9	145	9.9	
RACE (%)	N	%	N	%	0.500
Asian or Pacific Islander	30	2.05	30	2.05	
Black	200	13.65	215	14.68	
Hispanic	215	14.68	205	13.99	
Other	40	2.73	55	3.75	
White	980	66.89	960	65.53	
MEDIAN HOUSEHOLD INCOME (%)	N	%	N	%	0.863
<=51,999	440	30.03	435	29.69	
52K-65,999	435	29.69	420	28.67	
66K-87,999	325	22.18	330	22.53	
>=88k	265	18.09	280	19.11	
INSURANCE STATUS (%)	N	%	N	%	0.094
Medicaid	420	28.67	490	33.45	
Medicare	895	61.09	830	56.66	
Private Insurance	130	8.87	125	8.53	
HOSPITAL DIVISION (%)	N	%	N	%	<0.001
East North Central	265	18.09	265	18.09	
East South Central	55	3.75	35	2.39	
Middle Atlantic	255	17.41	235	16.04	
Mountain	60	4.1	115	7.85	
New England	105	7.17	95	6.48	

Pacific	170	11.6	195	13.31	
South Atlantic	270	18.43	215	14.68	
West North Central	95	6.48	135	9.22	
West South Central	190	12.97	175	11.95	
HOSPITAL BEDSIZE (%)	N	%	N	%	0.641
Large	685	46.76	690	47.1	
Medium	450	30.72	465	31.74	
Small	330	22.53	310	21.16	
HOSPITAL TEACHING STATUS (%)	N	%	N	%	0.205
Rural	170	11.6	145	9.9	
Urban nonteaching	285	19.45	270	18.43	
Urban teaching	1010	68.94	1050	71.67	
COMORBIDITIES (%)	N	%	N	%	P-value
Hypertension	410	27.99	460	31.4	0.043
Diabetes	160	10.92	160	10.92	0.999
Obesity	125	8.53	120	8.19	0.738
Drug misuse	20	1.37	**	**	0.066
Smoking	85	5.8	100	6.83	0.254
Alcohol	20	1.37	**	**	0.066
Chronic Pulmonary Disease	355	24.23	380	25.94	0.286
Hypothyroidism	190	12.97	245	16.72	0.004
Depression	85	5.8	100	6.83	0.254
Dementia	20	1.37	15	1.02	0.395

** Too small to report

Supplemental Table S2: Patient-level characteristics after propensity score matching for COVID-19-positive patients with cerebral palsy and Influenza-positive patients with cerebral palsy
